# Embracing Green Computing in Molecular Phylogenetics

**DOI:** 10.1093/molbev/msac043

**Published:** 2022-03-04

**Authors:** Sudhir Kumar

**Affiliations:** 1 Institute for Genomics and Evolutionary Medicine, Temple University, Philadelphia, PA, USA; 2 Department of Biology, Temple University, Philadelphia, PA, USA

**Keywords:** green computing, phylogenetics, molecular evolution, carbon footprint

## Abstract

Molecular evolutionary analyses require computationally intensive steps such as aligning multiple sequences, optimizing substitution models, inferring evolutionary trees, testing phylogenies by bootstrap analysis, and estimating divergence times. With the rise of large genomic data sets, phylogenomics is imposing a big carbon footprint on the environment with consequences for the planet’s health. Electronic waste and energy usage are large environmental issues. Fortunately, innovative methods and heuristics are available to shrink the carbon footprint, presenting researchers with opportunities to lower the environmental costs and greener evolutionary computing. Green computing will also enable greater scientific rigor and encourage broader participation in big data analytics.

Many biological disciplines apply computational approaches to investigate evolutionary questions involving the origins of genes, evolutionary relationships of organisms, positive and negative selection, the evolution of biodiversity, and genotype–phenotype connections across the tree of life. The importance of these questions is reflected by the escalating use of software for molecular evolutionary analyses (fig. 1). Paradoxically, the means by which we explore the tree of life actually negatively impact that evolving tree of life, because computing has environmental costs. A computers’ energy usage manifests into carbon dioxide emissions. Many scientists are seriously assessing the environmental cost of data analysis and the carbon footprint left by molecular evolutionary studies (Tao et al. 2019; Kumar and Sharma 2021; Álvarez-Carretero et al. 2022; Grealey et al. 2022). In particular, Grealey et al. (2022) have recently assessed the energy utilization and the associated carbon footprint of bioinformatics, including phylogenetic analysis and genome assembly.

**Fig. 1.**
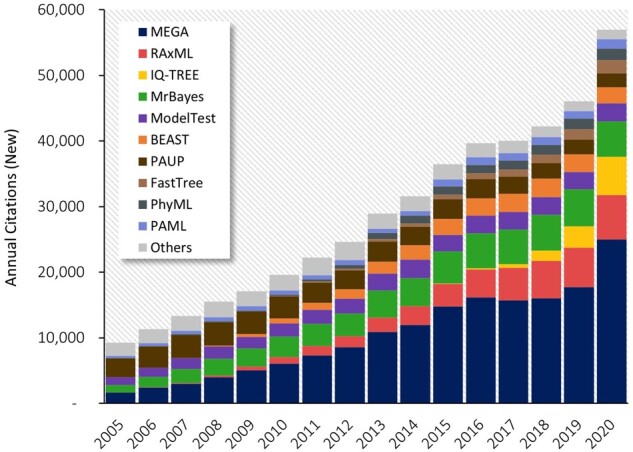
The use of computational methods in molecular evolution has been increasing quickly, as seen in the annual counts of new research articles citing the use of major software packages for molecular evolutionary and phylogenetic analyses. Citation counts for software packages were obtained from Google Scholar (last accessed January 25, 2022) for 2005–2020. See supplementary material, Supplementary Material online for more details on software versions included.

Strategies are being developed to achieve energy savings in a quest for greener computing in the sciences and a healthier global ecology with health benefits to the general public (Jones 2018; Portegies Zwart 2020; Stevens et al. 2020; Strubell et al. 2020; Bender et al. 2021; Lannelongue, Grealey, Bateman, et al. 2021; Grealey et al. 2022). For example, cloud computing avoids idle time, as partial CPU and memory use in standalone computers wastes energy (Shehabi et al. 2016; Jones 2018). However, speeding up research computing through faster processors and parallelization demands extra energy and, thus, emits more greenhouse gases. Using idle GPUs to assist CPUs can also result in greener computing, but this approach depends on appropriate software implementations (Grealey et al. 2022). Interestingly, energy production has a much smaller carbon footprint in some countries (e.g., Norway and Switzerland), making them better locations for cloud computing (Lannelongue, Grealey, and Inouye 2021).

Substantial reduction in energy costs can also be achieved by complementary means, which is the focus of this perspective. Here, I highlight conceptual and technical advances that can organically reduce computational time and memory of phylogenomics. I suggest that researchers choose methods, algorithms, and software practices that demand fewer compute cycles and less computer memory. These choices will diminish the carbon footprint of computational molecular evolution and be aligned with ecologically sound bioinformatic practices. These and future developments of resource-thrifty and accurate methods will amplify the impact of general strategies for greener computing.

## Carbon Footprints of Phylogenetic and Phylogenomic Analyses

A standard protocol in molecular phylogeny is first to assemble a set of sequences and subject them to alignment procedures to establish base-by-base homology across sequences from different species and genes (Kumar and Filipski 2007). The resulting multiple sequence alignments (MSAs) become ready for molecular phylogenetics after proper postprocessing, including manual curation (Yang and Rannala 2012; Kapli et al. 2020).

### Selecting the Optimal Model

In analyzing MSA, the usual first step is to estimate the substitution model that best describes the overall pattern of base changes. This analysis requires evaluating several models of nucleotide (or amino acid) substitution as well as models of rate variation across sites. Maximum likelihood (ML) tests of several nested and non-nested models under the Bayesian information criterion are frequently used. Model selection has a substantial carbon footprint for phylogenomic data sets.

For example, an MSA of 1.3 million base pairs from 37 mammalian species took 106 CPU hours and 9.3 gigabytes (GB) of peak memory in ModelFinder to select the optimal model (Kalyaanamoorthy et al. 2017). According to the Green Algorithms (GA) resource (Lannelongue, Grealey, and Inouye 2021), this analysis would require 1.6 kilowatt-hours (kWh) of energy and have a carbon footprint of 0.62 kgCO_2_e. GA suggests that a tree will take 20 days to scrub the environment of the greenhouse gasses emitted (table 1a1)! We can save more than 90% of the energy and, thus, emit less than 10% of the greenhouse gas by usingModelTest-NG (Darriba et al. 2020) and jModelTest (Posada 2008) that will produce similar results (table 1a). Recent machine-learning approaches also promise to provide green alternatives (Abadi et al. 2020; Burgstaller-Muehlbacher et al. 2021). Also, a machine-learning method for detecting autocorrelated evolutionary rates in a phylogeny (CorrTest; Tao et al. 2019) requires a small fraction of the energy used by a comparable Bayes factor analysis (table 1b).

**Table 1.** Carbon Footprints (gram CO_2_e) of Molecular Phylogenetic Analyses and Software for an MSA of 37 Mammalian Species and 1.3 Million Sites.

		Computer Resources			Environmental Impact	
		Time	Memory	Energy	C-footprint	Trees
Function	Method/Tool	(h)	(peak, MB)	(kWh)	(g)	(days)
(a) Optimal substitution model selection						
a1.	ModelFinder	106.0	9,300	1.64	617	20.1
a2.	jModelTest	8.8	3,700	0.12	44	1.5
a3.	ModelTest-NG	8.0	3,700	0.11	41	1.2
(b) Clock rate model selection						
b1.	Bayes factor	2,500.0	46,000	51.00	19,220	540.0
b2.	CorrTest	0.2	4,000	<0.01	1	<0.1
(c) Phylogeny inference						
c1.	Maximum likelihood	8.1	4,000	0.11	41	1.2
c2.	FastTree	0.7	700	0.01	3	0.1
c3.	Neighbor-joining	0.1	8	<0.01	<1	<0.1
(d) Statistical tests of phylogenies (ML)						
d1.	Standard bootstrap	980.0	3,100	13.00	4,850	159.0
d2.	Rapid bootstrap	98.0	3,700	1.00	493	16.2
d3.	Little bootstrap	18.9	100	0.23	86	2.7
d4.	Little+ultrafast-bootstraps	0.9	200	0.01	4	0.1
d5.	Bayesian	857.9	22,000	17.00	6,490	210.0
(e) Relaxed clock dating						
e1.	Bayesian (slow)	2,309.5	23,000	46.00	17,460	570.0
e3.	Bayesian (fast)	29.5	909	0.36	135	4.5
e3.	RelTime	0.1	8	<0.01	<1	<0.1

Note.—The C-footprint (Carbon footprint) is the amount (g) of CO_2_ released in the production of energy (kilowatt-hours, kWh) needed to power computers in the USA, estimated using the Green Algorithms website (Lannelongue, Grealey, and Inouye 2021). Tree days are calculated based on the information that a mature tree can scrub ∼917 g of CO_2_e per day (Grealey et al. 2022). The Supplementary Material online provides details on software used and the options applied.

### Building a Molecular Phylogeny

Using an MSA and the best-fit substitution model, we can make a phylogeny representing the evolutionary histories of genes and species. ML and minimum evolution (ME) are two widely used model-based optimality principles for reconstructing phylogenetic trees (Nei and Kumar 2000). The neighbor-joining method (Saitou and Nei 1987), based on the ME principle and used in thousands of studies, has a negligible carbon footprint (table 1c3) compared with popular heuristic searches under the ML optimality criterion (table 1c1). Another approach that combines optimality criteria (FastTree) has an intermediate environmental impact (table 1c2). The accuracy of phylogenies produced by different techniques is comparable for many applications (Rosenberg and Kumar 2001; Price et al. 2010; Yoshida and Nei 2016), so researchers have many excellent options for reducing the environmental impact of their analyses.

### Confidence Limits on Inferred Phylogenetic Groupings

Statistical evaluation of the robustness of inferred phylogenetic relationships is essential in evolutionary biology. Felsenstein’s (1985) bootstrap resampling has been the preferred approach, but it is computationally intensive, requiring the inference of hundreds of phylogenetic trees for pseudo-MSAs generated by sampling sites with replacement from the full data set. This analysis has a rather large carbon footprint (table 1d1), as does its Bayesian alternative that produces posterior probabilities for inferred evolutionary relationships (table 1d5). Many approximate energy-efficient methods are now available for phylogenomic data sets, including the technique Little Bootstraps (Sharma and Kumar 2021) for long sequences, and ultrafast bootstrapping (Minh et al. 2013) and Rapid bootstrapping (Stamatakis et al. 2008) for data sets containing large numbers of sequences. These approximate methods have much smaller carbon footprints than standard approaches (table 1d). Combining different techniques (Sharma and Kumar 2021) can save more than 99% in time, memory, and energy in testing the robustness of inferred phylogenies (table 1d4).

### From Phylogenies to Timetrees

Another common phylogenetic analysis is the estimation of divergence times corresponding to speciations, gene duplications, and the evolution of new strains. Relaxed clock methods have revolutionized this practice (Kumar and Hedges 2016; Tao et al. 2020). Bayesian and RelTime methods produce estimates of similar quality (e.g., Barba-Montoya et al. 2020; Mello et al. 2021), but their energy requirements are dramatically different (table 1e). There is also a large difference in the carbon footprints imposed by slow and fast Bayesian implementations (table 1e). Consequently, researchers have a large spectrum of more environmentally friendly alternatives for molecular dating methods.

### Green Software Implementations

Ultimately, efficient software implementation is the key to realizing the potential of all conceptional, methodological, and algorithmic innovations. The software design and resource utilization dictate energy consumption, so implementations that use less computer memory and time have a lower carbon footprint. Availability of software versions that can run on the cloud will also reduce carbon footprints. Another emerging area of improvement lies in creating stopping rules that can detect when further computing will not change the outcome significantly. For example, adaptive rules are being developed to automatically determine the number of bootstrap replicates needed for reliable confidence limits (Stamatakis 2014; Sharma and Kumar 2021). In the future, smarter software will avoid overcomputing, decreasing the carbon footprints of big data analyses.

## Benefits beyond Environmental Sustainability

Computationally efficient analyses will also enhance the rigor of scientific research, reducing the resources required to assess the robustness of inferences to subsetting of data, choice of substitution models and strategies, and combining multigene data sets. Computationally efficient and economical computing will encourage researchers to evaluate the reproducibility of published results. The currently high computational demands of reproducibility studies put efforts to reproduce research results out of the reach of researchers lacking access to high-performance computing infrastructure.

Greener computing is also a key to addressing equity, diversity, and sustainability in scientific research and education. Green computing requires fewer compute cycles and less computer memory. It reduces the expense of computational hardware and the cost of on-demand calculations. Economical computing makes computational research accessible to a broader community, as the research funding for scientific investigations is limited. Greener computing, therefore, will uniquely address economic disparities among researchers due to their local constraints. Greener alternatives for molecular phylogenetic analysis will increase participation by researchers worldwide in molecular evolutionary research and the genomic revolution in biology.

## Concluding Remarks

In the Anthropocene, where massive planetary changes are taking place because of human activity, computing is often thought of as a “clean” practice, when in fact, it can be quite the opposite. All branches of biology need to re-evaluate their practices in keeping with the underlying goal of studying life in the first place. For computational analyses, with the routine assembly of big data sets, analytical practices of the past hamper research by the need for excessive computing time and memory. These obstacles hinder both rigorous scientific investigations and wider participation in molecular phylogenetics. Large carbon footprints of many currently popular approaches have negative impacts on the environment, human health, and the sustainability of scientific computing. Fortunately, many accurate and resource-thrifty methods and algorithms are available for molecular phylogenetics. Applying these methods synergistically with computer hardware optimizations will help us achieve greater scientific rigor and broader participation while minimizing financial and environmental costs. I see a bright future for green computing in which conceptual and technical advances will further diminish the carbon footprints of increasingly complex phylogenomic analyses. 

## Supplementary Material

Supplementary information is available at *Molecular Biology and Evolution* online.

## Supplementary Material

msac043_Supplementary_Data

## References

[msac043-B1] Abadi S , Avram O, Rosset S, Pupko T, Mayrose I. 2020. Modelteller: model selection for optimal phylogenetic reconstruction using machine learning. Mol Biol Evol. 37(11):3338–3352. 32585030 10.1093/molbev/msaa154

[msac043-B2] Álvarez-Carretero S , Tamuri AU, Battini M, Nascimento FF, Carlisle E, Asher RJ, Yang Z, Donoghue PCJ, dos Reis M. 2022. A species-level timeline of mammal evolution integrating phylogenomic data. Nature 602(7896):263–267. 34937052 10.1038/s41586-021-04341-1

[msac043-B3] Barba-Montoya J , Tao Q, Kumar S. 2020. Using a GTR+Γ substitution model for dating sequence divergence when stationarity and time-reversibility assumptions are violated. Bioinformatics 36(Suppl_2):i884–i894. 33381826 10.1093/bioinformatics/btaa820 PMC7773479

[msac043-B4] Bender EM , Gebru T, McMillan-Major A, Shmitchell S. 2021. On the dangers of stochastic parrots: can language models be too big? In: FAccT ‘21: Proceedings of the 2021 ACM Conference on Fairness, Accountability, and Transparency. New York (NY): Association for Computing Machinery. p. 610–623.

[msac043-B5] Burgstaller-Muehlbacher S , Crotty SM, Schmidt HA, Drucks T, von Haeseler A. 2021. ModelRevelator: fast phylogenetic model estimation via deep learning. *bioRxiv*. Available from: https://www.biorxiv.org/content/10.1101/2021.12.22.473813v1. 10.1016/j.ympev.2023.107905 37595933

[msac043-B6] Darriba D , Posada D, Kozlov AM, Stamatakis A, Morel B, Flouri T. 2020. ModelTest-NG: a new and scalable tool for the selection of DNA and protein evolutionary models. Mol Biol Evol. 37(1):291–294. 31432070 10.1093/molbev/msz189 PMC6984357

[msac043-B7] Felsenstein J. 1985. Confidence limits on phylogenies: an approach using the bootstrap. Evolution 39(4):783–791. 28561359 10.1111/j.1558-5646.1985.tb00420.x

[msac043-B8] Grealey J , Lannelongue L, Saw W-Y, Marten J, Ruiz-Carmona S, Inouye M. 2022. The carbon footprint of bioinformatics. Mol Biol Evol. https://doi.org/10.1093/molbev/msac034. 10.1093/molbev/msac034 PMC8892942 35143670

[msac043-B9] Jones N. 2018. How to stop data centres from gobbling up the world’s electricity. Nature 561(7722):163–166. 30209383 10.1038/d41586-018-06610-y

[msac043-B10] Kalyaanamoorthy S , Minh BQ, Wong TKF, Von Haeseler A, Jermiin LS. 2017. ModelFinder: fast model selection for accurate phylogenetic estimates. Nat Methods. 14(6):587–589. 28481363 10.1038/nmeth.4285 PMC5453245

[msac043-B11] Kapli P , Yang Z, Telford MJ. 2020. Phylogenetic tree building in the genomic age. Nat Rev Genet. 21(7):428–444. 32424311 10.1038/s41576-020-0233-0

[msac043-B12] Kumar S , Filipski A. 2007. Multiple sequence alignment: in pursuit of homologous DNA positions. Genome Res. 17(2):127–135. 17272647 10.1101/gr.5232407

[msac043-B13] Kumar S , Hedges SB. 2016. Advances in time estimation methods for molecular data. Mol Biol Evol. 33(4):863–869. 26882983 10.1093/molbev/msw026 PMC5870647

[msac043-B14] Kumar S , Sharma S. 2021. Evolutionary sparse learning for phylogenomics. Mol Biol Evol. 38(11):4674–4682. 34343318 10.1093/molbev/msab227 PMC8557465

[msac043-B15] Lannelongue L , Grealey J, Bateman A, Inouye M. 2021. Ten simple rules to make your computing more environmentally sustainable. PLoS Comput Biol. 17:6–13. 10.1371/journal.pcbi.1009324 PMC8452068 34543272

[msac043-B16] Lannelongue L , Grealey J, Inouye M. 2021. Green algorithms: quantifying the carbon footprint of computation. Adv Sci (Weinh). 8(12):2100707–2100710. 34194954 10.1002/advs.202100707 PMC8224424

[msac043-B17] Mello B , Tao Q, Barba-Montoya J, Kumar S. 2021. Molecular dating for phylogenies containing a mix of populations and species by using Bayesian and RelTime approaches. Mol Ecol Resour. 21(1):122–136. 32881388 10.1111/1755-0998.13249 PMC8152102

[msac043-B18] Minh BQ , Nguyen MAT, Von Haeseler A. 2013. Ultrafast approximation for phylogenetic bootstrap. Mol Biol Evol. 30(5):1188–1195. 23418397 10.1093/molbev/mst024 PMC3670741

[msac043-B19] Nei M , Kumar S. 2000. Molecular evolution and phylogenetics. Oxford: Oxford University Press.

[msac043-B20] Portegies Zwart S. 2020. The ecological impact of high-performance computing in astrophysics. Nat Astron. 4(9):819–822.

[msac043-B21] Posada D. 2008. jModelTest: phylogenetic model averaging. Mol Biol Evol. 25(7):1253–1256. 18397919 10.1093/molbev/msn083

[msac043-B22] Price MN , Dehal PS, Arkin AP. 2010. FastTree 2—approximately maximum-likelihood trees for large alignments. PLoS One 5(3):e9490. 20224823 10.1371/journal.pone.0009490 PMC2835736

[msac043-B23] Rosenberg MS , Kumar S. 2001. Traditional phylogenetic reconstruction methods reconstruct shallow and deep evolutionary relationships equally well. Mol Biol Evol. 18(9):1823–1827. 11504861 10.1093/oxfordjournals.molbev.a003969

[msac043-B24] Saitou N , Nei M. 1987. The neighbor-joining method: a new method for reconstructing phylogenetic trees. Mol Biol Evol. 4(4):406–425. 3447015 10.1093/oxfordjournals.molbev.a040454

[msac043-B25] Sharma S , Kumar S. 2021. Fast and accurate bootstrap confidence limits on genome-scale phylogenies using little bootstraps. Nat Comput Sci. 1(9):573–577. 34734192 10.1038/s43588-021-00129-5 PMC8560003

[msac043-B26] Shehabi A , Smith SJ, Sartor DA, Brown RE, Herrlin M, Koomey JG, Masanet ER, Horner N, Azevedo IL, Lintner W. 2016. United States data center energy usage report. Berkeley (CA): Lawrence Berkeley National Laboratory.

[msac043-B27] Stamatakis A. 2014. RAxML version 8: a tool for phylogenetic analysis and post-analysis of large phylogenies. Bioinformatics 30(9):1312–1313. 24451623 10.1093/bioinformatics/btu033 PMC3998144

[msac043-B28] Stamatakis A , Hoover P, Rougemont J. 2008. A rapid bootstrap algorithm for the RAxML web servers. Syst Biol. 57(5):758–771. 18853362 10.1080/10635150802429642

[msac043-B29] Stevens ARH , Bellstedt S, Elahi PJ, Murphy MT. 2020. The imperative to reduce carbon emissions in astronomy. Nat Astron. 4(9):843–851.

[msac043-B30] Strubell E , Ganesh A, McCallum A. 2020. Energy and policy considerations for modern deep learning research. 34th AAAI Conf AAAI. 34(9):13693–13696.

[msac043-B31] Tao Q , Tamura K, Battistuzzi FU, Kumar S. 2019. A machine learning method for detecting autocorrelation of evolutionary rates in large phylogenies. Mol Biol Evol. 36(4):811–824. 30689923 10.1093/molbev/msz014 PMC6804408

[msac043-B32] Tao Q , Tamura K, Kumar S. 2020. Efficient methods for dating evolutionary divergences. In: Ho S, editor. The molecular evolutionary clock. Cham: Springer Nature. p. 197–219.

[msac043-B33] Yang Z , Rannala B. 2012. Molecular phylogenetics: principles and practice. Nat Rev Genet. 13(5):303–314. 22456349 10.1038/nrg3186

[msac043-B34] Yoshida R , Nei M. 2016. Efficiencies of the NJp, maximum likelihood, and Bayesian methods of phylogenetic construction for compositional and noncompositional genes. Mol Biol Evol. 33(6):1618–1624. 26929244 10.1093/molbev/msw042

