# Supplementary material for: Embracing Green Computing in Molecular Phylogenetics
Source: Mol Biol Evol. 2022 Mar 4;39(3):msac043. doi: 10.1093/molbev/msac043 (PMC8894743; doi:10.1093/molbev/msac043)
Supplement: msac043_Supplementary_Data [file msac043_supplementary_data.pdf]

Embracing Green computing in molecular phylogenetics — supplementary information

Citation impacts of software

We retrieved citation impacts from Google Scholar over the course of several years, beginning in 2012 with periodic updates with the final update made in January 2022 for the following software packages. MEGA (Kumar et al. 1994; Kumar et al. 2004; Kumar et al. 2005; Tamura et al. 2007; Kumar et al. 2008; Tamura et al. 2011; Kumar et al. 2012; Tamura et al. 2013; Kumar et al. 2016; Kumar et al. 2018; Stecher et al. 2020; Tamura et al. 2021), RAxML (Stamatakis et al. 2005; Stamatakis 2006; Stamatakis 2014; Kozlov et al. 2019), IQ-Tree (Minh et al. 2013; Nguyen et al. 2015; Trifinopoulos et al. 2016; Kalyaanamoorthy et al. 2017; Minh et al. 2020), MrBayes (Huelsenbeck and Ronquist 2001; Ronquist and Huelsenbeck 2003; Ronquist et al. 2012), ModelTest (Posada and Crandall 1998; Posada 2008; Darriba et al. 2012), BEAST (Drummond and Rambaut 2007; Drummond et al. 2012; Bouckaert et al. 2014), PAUP (Swofford 1993), FastTree (Price et al. 2009; Price et al. 2010), PhyML (Guindon et al. 2009; Guindon et al. 2010), PAML (Yang 1997; Yang 2007), Mesquite (Maddison and Maddison 2008), MacClade (Maddison and Maddison 2000), HyPHY (Kosakovsky Pond et al. 2005; Kosakovsky Pond and Frost 2005; Delport et al. 2010; Kosakovsky Pond et al. 2020), PHYLIP (Felsenstein 1993), and TREE-PUZZLE (Schmidt et al. 2002). To retrieve the yearly citation count for an article, the list of citations for each article was displayed on scholar.google.com, and then Google's custom range tool was used to list the number of citing papers for each year. Note that the counts presented indicate software tools' approximate impact and are not meant to convey the absolute total impact of computational molecular evolution. In the "Other" category, we pooled citation impacts for all the tools with fewer than 1000 citations in 2020.

Carbon footprint analysis

The nucleotide sequence alignment used consisted of 37 mammal species and 1,391,742 sites (Song et al. 2012). Carbon footprints of molecular phylogenetic analyses of this alignment were calculated using green-algorithms.org resource (v. 2.1) (Lannelongue et al. 2021), which was given information on computational time taken, peak memory used, the number of CPU cores applied, and the location of the computation (Pennsylvania, USA). The Real Usage Factor and Pragmatic Scaling Factor were set to no.

Substitution Model selection. For selecting the optimal substitution model, ModelFinder (Kalyaanamoorthy et al. 2017) and jModelTest (Posada 2008) implemented in IQ-TREE (Nguyen et al. 2015) were used. In ModelFinder, log-likelihoods were computed for 286

models along with a model of rate heterogeneity across sites. In jModelTest, 88 substitution models with equal or unequal base frequencies, a proportion of invariable sites, and rate variation among sites were tested. Also, used was ModelTest-NG (Darriba et al. 2020). All parameters were those set by default.

Clock model selection. The molecular clock model for relaxed clock dating was selected using MrBayes (Ronquist et al. 2012) and CorrTest (Tao et al. 2019). A Bayes factor analysis of the relaxed clock models implemented in MrBayes (Ronquist et al. 2012) with stepping-stone sampling was used. Two analyses were performed, one assuming autocorrelated rates (AR) among branches and the other assuming rates to vary independently (IR). The marginal likelihood calculation was used to estimate Bayes factors and posterior model probabilities. 50 steps of 40,000 MCMC iterations were used for each analysis, making 2,000,000 iterations including burn-in. These analyses could not be completed during the test period; thus, the carbon footprint was calculated by extrapolating the running time of 1,000 iterations to 2,000,000 iterations. The analysis using the AR model generated 1,000 iterations in 32 minutes, while the analysis using the IR model generated the same number of iterations in 43 minutes. The CorrTest analysis (Tao et al. 2019) was conducted in MEGA (Tamura et al. 2021). A tree with maximum likelihood (ML) estimates of branch length obtained in IQ-TREE using the GTR+Γ5 model was input to MEGA version 11 (Tamura et al. 2021). The final result includes the time and memory used in generating ML branch length estimates and conducting the CorrTest.

Phylogenetic inference. For IQ-TREE analysis (Nguyen et al. 2015), we used a GTR+Γ5 model, whereas a GTR+Γ20 model was used in FastTree2 (Price et al. 2010). The neighbor-joining method implemented in MEGA version 11 (Tamura et al. 2021) was used with pairwise distances estimated using the maximum composite likelihood analysis. A discrete Γ model with five rate categories (+Γ5) was assumed.

Confidence limits of phylogenetic inferences. Different ML tools and approaches were used in which the GTR+Γ4 model of nucleotide substitution was assumed. The standard bootstrap ML analysis was conducted in IQ-TREE (Nguyen et al. 2015), with the number of bootstrap replicates equal to 100. The rapid bootstrap (RAxML, Stamatakis et al. 2008) analysis also used the same options. The Little Bootstrap's time and memory needs and the combined Little + Ultra bootstrap results were obtained from (Sharma and Kumar 2021). The Bayesian analysis was conducted in MrBayes (Ronquist et al. 2012) using a GTR+Γ5 substitution model. Two MCMC chains were run, each consisting of 1,000,000 iterations, sampling every 200, with the burn-in set to 100,000 iterations. This analysis was not completed during the test period, so the total number of iterations required was determined by extrapolation. This analysis achieved a minimum ESS of 11 per 71,600 iterations after burn-in. Extrapolating the results with 1,301,818 iterations, we expect a minimum effective sample

size (ESS) of ~200. Then, if 1,000 iterations required 34m:20s of computation time, 1,301,818 iterations will require ~858 hours.

Timetree inference. Bayesian relaxed clock method implemented in MrBayes (Ronquist et al. 2012) was used with an AR clock model (*tk02*) and GTR+Γ5 nucleotide substitution model. The tree topology was fixed, and two calibrations were applied: one on the root (1.25,1.27) and another on the ingroup node (0.99,1.01). Diffuse gamma priors were applied to the mean rate (the gamma model is parametrized using two parameters: the mean and variance). The mean is assigned a lognormal hyperprior LN(0.125, 0.5), with the mean exp{0.125 + 0.52/2} = 1.0. The variance (*tk02varpr*) is assigned an exponential prior with a mean of 0.1. A uniform branching process to generate was used to generate the time prior. Two MCMC chains were run, each consisting of 1,000,000 iterations, sampling every 200, with the burn-in set to 100,000 iterations. This analysis did not finish during the test period, so the total number of iterations required was determined by extrapolation. This analysis achieved a minimum ESS of 5.7 per 90,200 iterations after burn-in. Extrapolating the results with 3,164,912 iterations, we expect to reach a minimum EES of ~200. Then, if 1,000 iterations required 43m:48s of computation time, 3,164,912 iterations will require ~2310 hours.

In MCMCTREE (Rannala and Yang 2007; Yang 2007), the approximate likelihood method (dos Reis and Yang 2011) was used to calculate the sequence likelihood for faster Bayesian relaxed clock dating analyses with the same substitution model and priors as above. A diffuse gamma prior was applied to the mean rate, μ ~ Γ(1, 1) and σ2 ~Γ(1,10). The birth and death prior were set to λ=μ=1, and ρ=0.0, which generates a uniform kernel density. We ran one MCMC chain with a burn-in of 250,000 iterations and a total of 20,000 samples collected every 100 iterations. This analysis achieved a minimum ESS of 8 per 2,000,000 iterations, extrapolating the results. With 50,000,000 iterations, we expect a minimum EES of ~200. Thus, if 2,250,000 iterations required 41m:15s of computation time, then 50,000,000 iterations will require 15h:20m. Moreover, the Hessian matrix required 14h:8m, so the total time of the MCMCTREEE analysis was 29h:28m.

Finally, the RelTime analysis with Ordinary Least-Squares estimates of branch length was conducted in MEGA version 11. A maximum composite likelihood model and a discrete Gamma model with five rate categories were applied. Only one calibration (0.99,1.01) was used on the ingroup node.

References

Bouckaert R, Heled J, Kühnert D, Vaughan T, Wu CH, Xie D, Suchard MA, Rambaut A, Drummond AJ. 2014. BEAST 2: A Software Platform for Bayesian Evolutionary Analysis. *PLoS Comput. Biol.* 10:1–6.

Darriba D, Posada D, Kozlov AM, Stamatakis A, Morel B, Flouri T. 2020. ModelTest-NG: A New and Scalable Tool for the Selection of DNA and Protein Evolutionary Models. *Mol. Biol. Evol.* 37:291–294.

Darriba D, Taboada GL, Doallo R, Posada D. 2012. JModelTest 2: More models, new heuristics and parallel computing. *Nat. Methods* 9:772.

Delport W, Poon AFY, Frost SDW, Kosakovsky Pond SL. 2010. Datamonkey 2010: A suite of phylogenetic analysis tools for evolutionary biology. *Bioinformatics* 26:2455–2457.

Drummond AJ, Rambaut A. 2007. BEAST: Bayesian evolutionary analysis by sampling trees. *BMC Evol. Biol.* 7:214.

Drummond AJ, Suchard MA, Xie D, Rambaut A. 2012. Bayesian phylogenetics with BEAUti and the BEAST 1.7. *Mol. Biol. Evol.* 29:1969–1973.

Felsenstein J. 1993. PHYLIP (Phylogeny Inference Package) version 3.5c. Distributed by the author. Department of Genetics, University of Washington, Seattle, M.A.

Guindon S, Delsuc F, Dufayard JF, Gascuel O. 2009. Estimating maximum likelihood phylogenies with PhyML. In: Posada D, editor. Bioinformatics for DNA Sequence. New York: Humana Press. p. 113–137.

Guindon S, Dufayard JF, Lefort V, Anisimova M, Hordijk W, Gascuel O. 2010. New algorithms and methods to estimate maximum-likelihood phylogenies: Assessing the performance of PhyML 3.0. *Syst. Biol.* 59:307–321.

Huelsenbeck JP, Ronquist F. 2001. MRBAYES: Bayesian inference of phylogenetic trees. *Bioinformatics* 17:754–755.

Kalyaanamoorthy S, Minh BQ, Wong TKF, Von Haeseler A, Jermiin LS. 2017. ModelFinder: Fast model selection for accurate phylogenetic estimates. *Nat. Methods* 14:587–589.

Kosakovsky Pond SL, Frost SDW. 2005. Datamonkey: Rapid detection of selective pressure on individual sites of codon alignments. *Bioinformatics* 21:2531–2533.

Kosakovsky Pond SL, Frost SDW, Muse S V. 2005. HyPhy: Hypothesis testing using phylogenies. *Bioinformatics* 21:676–679.

Kosakovsky Pond SL, Poon AFY, Velazquez R, Weaver S, Hepler NL, Murrell B, Shank SD, Magalis BR, Bouvier D, Nekrutenko A, et al. 2020. HyPhy 2.5 - A Customizable Platform for Evolutionary Hypothesis Testing Using Phylogenies. *Mol. Biol. Evol.* 37:295–299.

Kozlov AM, Darriba D, Flouri T, Morel B, Stamatakis A. 2019. RAxML-NG: A fast, scalable and user-friendly tool for maximum likelihood phylogenetic inference. *Bioinformatics* 35:4453–4455.

Kumar, S., K. T, Nei M. 2004. MEGA3: Integrated software for Molecular Evolutionary Genetics. *Brief. Bioinform.* 5:150–163.

Kumar S, Nei M, Dudley J, Tamura K. 2008. MEGA: A biologist-centric software for evolutionary analysis of DNA and protein sequences. *Brief. Bioinform.* 9:299–306.

Kumar S, Stecher G, Li M, Knyaz C, Tamura K. 2018. MEGA X: Molecular evolutionary genetics analysis across computing platforms. *Mol. Biol. Evol.* 35:1547–1549.

Kumar S, Stecher G, Peterson D, Tamura K. 2012. MEGA-CC: Computing core of molecular

evolutionary genetics analysis program for automated and iterative data analysis. *Bioinformatics* 28:2685–2686.

Kumar S, Stecher G, Tamura K. 2016. MEGA7: Molecular Evolutionary Genetics Analysis Version 7.0 for Bigger Datasets. *Mol. Biol. Evol.* 33:1870–1874.

Kumar S, Tamura K, Jakobsen IB, Nei M. 2005. MEGA2: molecular evolutionarygenetics analysis software. *Bioinformatics* 21:3572–3574.

Kumar S, Tamura K, Nei M. 1994. MEGA: Molecular Evolutionary Genetics Analysis software for microcomputer. *Comput. Appl. Biosci.* 10:189–191.

Lannelongue L, Grealey J, Inouye M. 2021. Green Algorithms: Quantifying the Carbon Footprint of Computation. *Adv. Sci.* 8:1–10.

Maddison DR, Maddison WP. 2000. MacClade 4: Analysis of phylogeny and character evolution. Version 4.0. Sunderland, Massachusetts: Sinauer Associates

Maddison WP, Maddison DR. 2008. Mesquite: a modular system for evolutionary analysis. *Evolution.* 62:1103–1118.

Minh BQ, Nguyen MAT, Von Haeseler A. 2013. Ultrafast approximation for phylogenetic bootstrap. *Mol. Biol. Evol.* 30:1188–1195.

Minh BQ, Schmidt HA, Chernomor O, Schrempf D, Woodhams MD, Von Haeseler A, Lanfear R, Teeling E. 2020. IQ-TREE 2: New Models and Efficient Methods for Phylogenetic Inference in the Genomic Era. *Mol. Biol. Evol.* 37:1530–1534.

Nguyen LT, Schmidt HA, Von Haeseler A, Minh BQ. 2015. IQ-TREE: A fast and effective stochastic algorithm for estimating maximum-likelihood phylogenies. *Mol. Biol. Evol.* 32:268–274.

Posada D. 2008. jModelTest: Phylogenetic model averaging. *Mol. Biol. Evol.* 25:1253–1256.

Posada D, Crandall KA. 1998. MODELTEST: testing the model of DNA substitution David. *Bioinformatics* 14:817–818.

Price MN, Dehal PS, Arkin AP. 2009. Fasttree: Computing large minimum evolution trees with profiles instead of a distance matrix. *Mol. Biol. Evol.* 26:1641–1650.

Price MN, Dehal PS, Arkin AP. 2010. FastTree 2 - Approximately maximum-likelihood trees for large alignments. *PLoS One* 5:e9490.

Rannala B, Yang Z. 2007. Inferring speciation times under an episodic molecular clock. *Syst. Biol.* 56:453–466.

dos Reis M, Yang Z. 2011. Approximate likelihood calculation on a phylogeny for Bayesian Estimation of Divergence Times. *Mol. Biol. Evol.* 28:2161–2172.

Ronquist F, Huelsenbeck JP. 2003. MrBayes 3: Bayesian phylogenetic inference under mixed models. *Bioinformatics* 19:1572–1574.

Ronquist F, Teslenko M, Van Der Mark P, Ayres DL, Darling A, Höhna S, Larget B, Liu L, Suchard MA, Huelsenbeck JP. 2012. Mrbayes 3.2: Efficient bayesian phylogenetic inference and model choice across a large model space. *Syst. Biol.* 61:539–542.

Schmidt HA, Strimmer K, Vingron M, Von Haeseler A. 2002. TREE-PUZZLE: Maximum likelihood phylogenetic analysis using quartets and parallel computing. *Bioinformatics* 18:502–504.

Sharma S, Kumar S. 2021. Fast and accurate bootstrap confidence limits on genome-scale phylogenies using little bootstraps. *Nat. Comput. Sci.* 1:573–577.

Song S, Liu L, Edwards S V, Wu S. 2012. Resolving conflict in eutherian mammal phylogeny using phylogenomics and the multispecies coalescent model. *Proc. Natl. Acad. Sci. U.*

S. A. 109:14942–1497.

Stamatakis A. 2006. RAxML-VI-HPC: Maximum likelihood-based phylogenetic analyses with thousands of taxa and mixed models. *Bioinformatics* 22:2688–2690.

Stamatakis A. 2014. RAxML version 8: A tool for phylogenetic analysis and post-analysis of large phylogenies. *Bioinformatics* 30:1312–1313.

Stamatakis A, Hoover P, Rougemont J. 2008. A rapid bootstrap algorithm for the RAxML web servers. *Syst. Biol.* 57:758–771.

Stamatakis A, Ludwig T, Meier H. 2005. RAxML-III: A fast program for maximum likelihood-based inference of large phylogenetic trees. *Bioinformatics* 21:456–463.

Stecher G, Tamura K, Kumar S. 2020. Molecular Evolutionary Genetics Analysis (MEGA) for macOS. *Mol. Biol. Evol.* 37:1237–1239.

Swofford DL. 1993. PAUP: phylogenetic analysis using parsimony (Computer program and manual). Distributed by the author. Florida Museum of Natural History, University of Florida. Gainesville, FL.

Tamura K, Dudley J, Nei M, Kumar S. 2007. MEGA4: Molecular Evolutionary Genetics Analysis (MEGA) software version 4.0. *Mol. Biol. Evol.* 24:1596–1599.

Tamura K, Peterson D, Peterson N, Stecher G, Nei M, Kumar S. 2011. MEGA5: Molecular evolutionary genetics analysis using maximum likelihood, evolutionary distance, and maximum parsimony methods. *Mol. Biol. Evol.* 28:2731–2739.

Tamura K, Stecher G, Kumar S. 2021. MEGA11: Molecular Evolutionary Genetics Analysis version 11. *Mol. Biol. Evol.* 38:3022–3027.

Tamura K, Stecher G, Peterson D, Filipski A, Kumar S. 2013. MEGA6: Molecular evolutionary genetics analysis version 6.0. *Mol. Biol. Evol.* 30:2725–2729.

Tao Q, Tamura K, Battistuzzi FU, Kumar S. 2019. A machine learning method for detecting autocorrelation of evolutionary rates in large phylogenies. *Mol. Biol. Evol.* 36:811–824.

Trifinopoulos J, Nguyen LT, von Haeseler A, Minh BQ. 2016. W-IQ-TREE: a fast online phylogenetic tool for maximum likelihood analysis. *Nucleic Acids Res.* 44:W232–W235.

Yang Z. 1997. PAML: A program package for phylogenetic analysis by maximum likelihood. *Bioinformatics* 13:555–556.

Yang Z. 2007. PAML 4: Phylogenetic analysis by maximum likelihood. *Mol. Biol. Evol.* 24:1586–1591.
